# Internet-based treatment for adults with depressive symptoms: the protocol of a randomized controlled trial

**DOI:** 10.1186/1471-244X-7-72

**Published:** 2007-12-19

**Authors:** Lisanne Warmerdam, Annemieke van Straten, Pim Cuijpers

**Affiliations:** 1 Department of Clinical Psychology, Institute for Research in Extramural Medicine, VU University, Amsterdam, The Netherlands

## Abstract

**Background:**

Depression is a highly prevalent condition, affecting more than 15% of the adult population at least once in their lives. Guided self-help is effective in the treatment of depression. The purpose of this study is to investigate the effectiveness of two Internet-based guided self-help treatments with adults reporting elevated depressive symptoms. Other research questions concern the identification of potential mediators and the search for subgroups who respond differently to the interventions.

**Methods:**

This study is a randomized controlled trial with three conditions: two treatment conditions and one waiting list control group. The two treatment conditions are Internet-based cognitive behavior therapy and Internet-based problem-solving therapy. They consist of 8 and 5 weekly lessons respectively. Both interventions are combined with support by e-mail. Participants in the waiting list control group receive the intervention three months later.

The study population consists of adults from the general population. They are recruited through advertisements in local and national newspapers and through banners on the Internet. Subjects with symptoms of depression (≥ 16 on the Center for Epidemiological Studies Depression scale) are included. Other inclusion criteria are having sufficient knowledge of the Dutch language, access to the Internet and an e-mail address.

Primary outcome is depressive symptoms. Secondary outcomes are anxiety, quality of life, dysfunctional cognitions, worrying, problem solving skills, mastery, absence at work and use of healthcare. We will examine the following variables as potential mediators: dysfunctional cognitions, problem solving skills, worrying, anxiety and mastery. Potential moderating variables are: socio-demographic characteristics and symptom severity. Data are collected at baseline and at 5 weeks, 8 weeks, 12 weeks and 9 months after baseline. Analyses will be conducted on the intention-to-treat sample.

**Discussion:**

This study evaluates two Internet-based treatments for depression, namely cognitive behavioral therapy and problem-solving therapy. The effectiveness of Internet-based problem-solving therapy suggest that this may be a worthwhile alternative to other more intensive treatment options. Strengths and limitations of this study are discussed.

**Trial registration:**

Current Controlled Trials ISRCTN16823487

## Background

Currently, depressive disorders are the fourth disorder worldwide in terms of disease burden, and will be the disorder with the highest disease burden in high-income countries by 2030 [1]. Depressive disorders are highly prevalent, affecting more than 15% of the general population once in their lives [2]. Depressive disorders are associated with substantial losses in quality of life in patients and their relatives [3], with increased mortality rates [4,5], with high levels of service use, and with huge economic costs [6,7]. Comorbidity is high among depressive disorders, especially with anxiety disorders and alcohol abuse [2].

In the last two decades, many studies evaluated self-help interventions as a treatment for depression. Guided self-help can be described as a standardized psychological treatment in which a patient can help himself, with only minimal support from a (professional) therapist. The form in which guided self-help is presented consists of books, videos or computer programs. Several studies have found that guided self-help is effective for the treatment of minor-to-moderate depression [8,9], including Internet-based self-help [10] and meta-analyses have found that the effects of self-help interventions are comparable to those of traditional psychological treatments [11,12]. Some support during the Internet intervention has been shown to be important. A meta-analysis of Internet-based cognitive behavior therapy (CBT) for symptoms of depression and anxiety shows that interventions with therapist support had a large mean effect size while interventions without therapist support had a small mean effect size [12].

Internet-based self-help is interesting for several reasons. Besides advantages such as low costs and high efficiency, the lack of sufficiently skilled therapists makes self-help approaches attractive [11]. Furthermore, the Internet offers the possibility of prompt feedback, monitoring and presentation of material on a step-by-step basis [13]. Another important reason why Internet-based self-help is interesting is that this form of self-help can probably reach many depressed persons who cannot be reached with traditional forms of therapy. Studies carried out in Canada or Europe reported high percentages of undertreatment for adults with depression varying from 36% to 43% [14,15]. Many depressed persons who won't go to a professional therapist have objections like 'talking' does not help, lack of willingness to talk to a stranger about personal problems, fear of a stigma [11]. The low-threshold accessibility of the Internet makes it suitable for offering and receiving help for psychological problems.

Most self-help therapies are developed for treating specific disorders like depression or a specific anxiety disorder. Furthermore, most self-help therapies are based on CBT because face-to-face CBT has proven to be effective [16] and because its structured format makes it very suitable for self-help purposes. However, it is unknown whether CBT self-help is really more effective than other self-help formats. Problem-solving therapy (PST) may be effective in several problem areas. Face-to-face PST is effective in reducing depression and in several other mental health problems [17,18]. As far as we know, there is no study which compares Internet-based CBT with an Internet-based generic intervention like PST, for depression.

However, problem-solving therapies have not been adapted for use through the Internet, so we developed a new, generic method for multiple mental health problems that could be applied through the Internet. As a general framework for the intervention, we used the model developed by Bowman and colleagues [19,20], which is called Self-Examination Therapy (SET), and we combined it with cognitive behavioral components and problem-solving procedures. The general idea of SET is that subjects learn to regain control over their problems and lives by (1) determining what really matters to them (2) investing energy only in those problems that are related to what matters (3) thinking less negatively about the problems that are unrelated and (4) accepting those situations that cannot be changed. This method has been found to be effective in several studies in the United States [19-21]. In a previous study we have shown the effectiveness of our course in patients with different mental health symptoms [22]. The comparison between CBT and PST is interesting for another reason. Our PST-based intervention takes only five weeks (CBT in this study takes eight weeks) and may be a worthwhile alternative to other more intensive treatment options.

While there is consensus about the effectiveness of guided self-help for depression, it is still unknown how these interventions work and for whom they work. This holds true for other types of intervention as well. In this study, we examine potential mediating and moderating variables. The main objective of this study is to evaluate the effectiveness of two Internet-based guided self-help treatments with adults reporting elevated depressive symptoms comparing to a waiting list control group. The evaluated self-help treatments are CBT and PST. The secondary objective of this study is to examine potential mediators and moderators. In this article we describe the design of the study.

## Methods

### Study design

This study is a randomized controlled trial. Subjects were randomized into three groups: two Internet-based self-help interventions (CBT and PST) and a waiting list control group. The study protocol, information brochure and informed consent form were approved by the Medical Ethics Committee of the VU University Medical Center (registration number 2006/168).

### Inclusion and exclusion criteria

All adults aged 18 years and older with depressive symptoms who are willing to perform a self-help course, were eligible for this study. Inclusion criteria were: having elevated depressive symptoms (scoring above the cut-off score of 16 or more on the Center of Epidemiologic Studies Depression – scale (CES-D), sufficient knowledge of the Dutch language, access to Internet and having an e-mail address. No exclusion criteria were defined for this study.

### Recruitment

Subjects for this study were recruited through advertisements in daily and weekly newspapers and via the Internet. This was done in a period of 5 weeks. Application took place via a website. After application, subjects received a brochure about this study and an informed consent form by post. After giving informed consent, subjects received the baseline questionnaire by e-mail. Subjects with a score of 16 or higher on the CES-D were included. Subjects with more severe symptoms of depression (indicated by a CES-D score of 32 or higher) were advised to consult their general practitioner, but could participate in this study.

### Randomization

Randomization took place at an individual level after the baseline measurement and one week before the start of the interventions. Subjects were randomized into three groups – two intervention groups and a waiting list control group. Received baseline questionnaires were numbered in order of arrival. We used block randomization, with each block containing 9 allocations. An independent researcher made the allocation schedule with a computerized random number generator. A week before the start of the interventions, subjects were informed about the randomization outcome.

### Interventions

#### Problem-Solving Treatment

Our PST- intervention is based on SET [19]. It has been expanded with more information, examples, exercises and forms. The theoretical assumption underpinning problem-solving is that psychological symptoms of depression and anxiety are often caused by practical problems people face in their daily lives. Hence, if people's problems can be resolved, their symptoms will improve. During PST, an individual learns a specific problem-solving procedure in an attempt to resolve their problems in a structured way [23].

PST in this study consisted of three steps. First the subjects described what really matters to them. Second they wrote down their current worries and problems. They categorised these problems into three categories (a) unimportant problems (problems unrelated to the things that matter to them), (b) problems which can be solved, and (c) problems which cannot be solved (e.g. the loss of a loved one). For each of these three types of problems a different strategy is proposed to solve the problems or to learn to cope with the unsolvable ones. A great amount of time is spent on solvable problems by using a six steps procedure, namely describing the problem, brainstorming solutions, choosing the best solution, making a plan for carrying out the solution, actually carrying out the solution and evaluation. During the third and last step, the subjects made a plan for the future in which they described how they will try to accomplish those things that matter most to them. The course took five weeks, with one lesson a week. Every week the participants were asked to make exercises and to send these to their coach.

#### Cognitive Behavioral Therapy

The Internet-based CBT intervention was developed by the Trimbos Institute, The Netherlands Institute of Mental Health and Addiction. This intervention is based on the "Coping with Depression" course (CWD) [24], Dutch version [25]. CWD is a highly structured psycho-educational form of cognitive behavior therapy for depression. Theoretically, this course is based on the social learning theory according to which depression is associated with a decrease in pleasant and an increase in unpleasant person-environment interactions. People's problems are viewed as behavioral and cognitive patterns which can be unlearned or relearned. The course was designed to provide training in skills which can be used to change these behavioral and cognitive patterns [24]. The cognitive skills are based on the cognitive therapy by Beck and his colleagues [26] and the rational emotive therapy from Ellis. The pleasant events approach has been developed by Lewinsohn and colleagues [27].

Like CWD, CBT in this study included psycho-education and focused on skills such as relaxation, cognitive restructuring (including worrying), social skills and how to increase the number of pleasant events. The intervention made use of text, exercises, audio and video fragments. CBT consisted of 8 lessons, one lesson a week. Twelve weeks later, the 9th lesson took place.

#### Waiting-list control group

Subjects on the waiting list received no treatment or support but started the intervention three months after the intervention group ended. Subjects on the waiting list received problem solving treatment.

#### Support

Subjects in both intervention groups received support during the intervention period by email from Master students in clinical psychology. Prior to the start of the interventions, they received training of 6 hours in total. This training was given by the first author of this article. First, the students read the intervention material themselves, then they carried out the assignments, and practised giving feedback to each other by e-mail. They also practised with case material and their feedback was discussed.

Support was directed at guiding the participant through the intervention, not at depressive symptoms or other problems. Every week, a standardized e-mail was sent to the participants. This e-mail communicated the lesson of that week and the date on which the assignments were to be sent to their coach. When participants sent their assignments to their coach, they received feedback within three working days. All feedback was checked by the first author and if necessary provided with comments before it was sent to the participants.

### Assessments

Five assessments took place. The first assessment (baseline) took place before the start of the intervention. Other assessments took place at 5, 8, 12 weeks and 9 months after baseline.

### Instruments

#### Primary outcome

##### Depressive symptoms

Symptoms of depression were measured with the Center for Epidemiological Studies Depression scale (CES-D) [28]. This questionnaire is widely used for identifying people with depressive symptomatology. Its validity has been tested in different populations [29-31]. The CES-D consists of 20 items and the total score varies between 0 and 60. Scores of 16 and higher represent a clinically significant level of depressive symptoms [28]. The cut-off score of 16 was used in this study as an inclusion criterion.

#### Secondary outcomes

##### Anxiety symptoms

The anxiety subscale of the Hospital Anxiety and Depression Scale (HADS) was used for the measurement of anxiety symptoms [32]. The anxiety subscale consists of 7 items. Scores range from 0 to 21 with higher scores indicating more anxiety. The HADS showed good homogeneity and reliability, with Cronbach's alpha ranging from .81 to .84 in different normal and clinical Dutch samples [32].

##### Quality of life

Quality of life was assessed with the EuroQol Questionnaire (EQ5D) [33], which is a validated tool for measuring general health-related quality of life. It consists of five items (mobility, self-care, usual activities, pain/discomfort and anxiety/depression), each of which is rated as causing 'no problems', 'some problems' or 'extreme problems'. The EQ5D thus distinguishes 486 unique health states. Each unique health state has a utility score which ranges from 0 (poor health) to 1 (perfect health). We used this single EQ5D summary index score.

##### Dysfunctional cognitions

The Dysfunctional Attitudes Scale (DAS) is a 40-item self-report measure designed to assess cognitive vulnerability to depression [34]. The DAS is one of the most widely used questionnaires to measure cognitions in relation to depression. The Dutch version of the DAS showed good reliability and satisfactory validity [35].

##### Health care utilisation

We used the Trimbos and iMTA Questionnaire on Costs Associated with Psychiatric Illness (TiC-P) [36] to collect data on direct and indirect costs from the participants. The first part of the Tic-P measures the amount of medical care received by the participants, the second part measures work productivity.

##### Worrying

Worrying was measured by the Penn State Worry Questionnaire (PSWQ). The PSWQ [37] is a 16-item self-report inventory. Scores range from 16 to 80 with higher scores representing more worry. De PSWQ showed good reliability in normal and clinical populations [38].

##### Problem-Solving skills

The Social Problem-Solving Inventory-Revised (SPSI-R) which is designed by D'Zurilla, was used for measuring problem solving skills. This questionnaire was designed to measure people's ability to resolve problems of everyday living. The SPSI-R contains 52 items and consists of the following five scales: Positive problem Orientation (PPO), Negative Problem Orientation (NPO), Rational Problem Solving (RPS), Impulsivity/Carelessness Style (ICS) and Avoidance Style (AS). Alphas for these five scales ranged from .76 to .92 and test-retest reliability ranged from .72 to .88 [39].

##### Perceived control

We assessed perceived control with the Mastery Scale [40]. This scale has 7 items regarding how much an individual perceives having control over things in his or her life. Items are rated on a 4-point scale with higher scores indicating greater perceived control. The questionnaire has good psychometric properties [40].

### Sample size

The sample size of this study was based on the expected difference on the primary outcome variable, i.e. depressive symptoms, between the intervention groups and the waiting list control group at post-test. Based on a power of 0.80 in a one-tailed test, an alpha of 0.05, we needed 100 subjects in each condition to show an effect-size of 0.40. Therefore, the total sample size was determined at 300.

### Statistical analysis

Analyses were performed according to the intention-to-treat principle. Overall treatment efficacy was performed with linear mixed modeling analysis using SPSS. General growth mixture modeling was used for the identification of subgroups in the sample and for analyzing mediating variables, using M-*plus*. With this method, it is possible to identify distinct groups of individuals, differing in the initial level and course of a specific behavior, through the empirical identification of developmental trajectories [41]. The advantage of this method is that the identification of subpopulations within a sample is based on the target behavior itself (in our case depressive symptoms over time). This technique also makes it possible to examine whether the effects of interventions differ for different categories of patients, to ascertain which characteristics (moderators) predict membership of one of these categories, and to establish whether outcomes are different for each category [42]. The following variables were examined for their mediating role: dysfunctional cognitions, problem solving skills, worrying, anxiety and mastery. The moderators we studied that may affect the efficacy of both treatments were: socio-demographic characteristics and severity of symptoms.

## Discussion

This study compares two Internet-based guided self-help interventions, i.e. CBT and PST. It is interesting to know whether the interventions result in a decline of depressive symptoms compared to a waiting list control group and what the difference is between the two interventions. The effectiveness of Internet-based PST suggests that this may be a viable alternative in the treatment of depression because of its brevity and flexibility. This study provides more insight into the effectiveness of Internet-based guided self-help, thereby making a contribution to the development of Internet interventions that are accessible to a large population. A discussion of some methodological issues of this study follows below.

We chose to recruit people by advertising in some daily and regional newspapers and via the Internet. This may lead to selection-bias and the results may probably not be generalisable to all depressed people. On the other hand, effective Internet-based self-help is probably most interesting for people who look for other forms of health care instead of going to a general practioner or psychologist for instance.

The comparison of the two interventions in our study may lead to some methodological difficulties because of the different length of both interventions. It can be argued that differences in effect have to do with the time component. Nevertheless, the comparison can give insight into the necessary duration of an intervention to lead to a reduction of depressive symptoms and after how many weeks this reduction is reached.

A strong aspect of the design of this study is the number of measurements. We made use of five measurements thereby making it possible to analyze the development of different kind of symptoms over time. Of particular interest is the second measurement, after five weeks, which makes it possible to look for mediating variables in Internet-based CBT.

A limitation of this study concerns the (lack of) diagnosis. Subjects are included on the basis of a self-rating instrument, not on the basis of a diagnostic interview. It is unknown whether the subjects in our study meet the criteria for a depressive disorder. Therefore, we lack the gold standard for diagnosing, and this makes comparison with other studies difficult. The absence of diagnosing in this study has to do with our intention to make this kind of Internet-based self-help interventions applicable and accessible for a broad population with clinically relevant depressive symptoms. If these interventions turn out to be effective for people from the community it may be possible in the future to allow people to follow Internet-based self-help courses on their own without the need for a professional therapist. From an economic perspective, this idea is attractive because costs are saved which could for example be used for patients with more extensive care needs.

For practical reasons, this trial was conducted in two parts. The first part started in September 2006 and the second part in January 2007. The data of both parts are taken together. It is possible that some influences like the time of year may have biased the results. Therefore, the data were analyzed separately for both parts and combined afterwards.

## Competing interests

The author(s) declare that they have no competing interests.

## Authors' contributions

PC obtained funding for the study. All authors contributed to the design of this study and the making of the Internet-based PST-intervention. LW supervised the students who provided e-mail support for the participants. She also took care of the recruitment of participants and data collection. LW drafted the manuscript. All authors contributed to the further writing of the manuscript. All authors read and approved the final manuscript.

## Pre-publication history

The pre-publication history for this paper can be accessed here:


